# Global Status of* Porcine circovirus* Type 2 and Its Associated Diseases in Sub-Saharan Africa

**DOI:** 10.1155/2017/6807964

**Published:** 2017-03-12

**Authors:** Kayode O. Afolabi, Benson C. Iweriebor, Anthony I. Okoh, Larry C. Obi

**Affiliations:** ^1^SAMRC Microbial Water Quality Monitoring Centre, University of Fort Hare, Private Bag X1314, Alice 5700, Eastern Cape, South Africa; ^2^Applied and Environmental Microbiology Research Group (AEMREG), Department of Biochemistry and Microbiology, University of Fort Hare, Private Bag X1314, Alice 5700, Eastern Cape, South Africa; ^3^Academic and Research Division, University of Fort Hare, Private Bag X1314, Alice, Eastern Cape, South Africa

## Abstract

Globally,* Porcine circovirus* type 2 (PCV2) is a recognized viral pathogen of great economic value in pig farming. It is the major cause of ravaging postweaning multisystemic wasting syndrome (PMWS) and many other disease syndromes generally regarded as* Porcine circovirus* associated diseases (PCVAD) in Europe. PCV2 infections, specifically PMWS, had impacted huge economic loss on swine production at different regions of the world. It has been studied and reported at different parts of the globe including: North and South America, Europe, Asia, Oceania, Middle East, and the Caribbean. However, till date, this virus and its associated diseases have been grossly understudied in sub-Sahara African region and the entire continent at large. Two out of forty-nine, representing just about 4% of countries that make up sub-Sahara Africa presently, have limited records on reported cases and occurrence of the viral pathogen despite the ubiquitous nature of the virus. This review presents an overview of the discovery of* Porcine circovirus* and its associated diseases in global pig herds and emphasizes the latest trends in PCV2 vaccines and antiviral drugs development and the information gaps that exist on the occurrence of this important viral pathogen in swine herds of sub-Saharan Africa countries. This will serve as wake-up call for immediate and relevant actions by stakeholders in the region.

## 1. Introduction

Pig rearing is one of the fastest growing livestock sector worldwide [1] as it is a valuable source of animal protein globally and the industry contributes largely to the economy of many countries [2]. Despite the huge economic potentials of piggery business, many farms are faced with myriads of problems orchestrated by diseases that have the capacity to decimate herds. In as much as many opportunities abound in pig rearing especially for small scale farmers, their efforts to improve on their production capacity are hampered by great loss of animals to diseases [3]. Furthermore, the problem becomes more complicated by limited information on the relative frequency of occurrence of the different diseases and their detrimental effects on pig production, most especially in developing countries of Africa [4]. Swine diseases of economic importance range between bacterial, viral, fungal, and protozoan origins. According to Vidigal et al. [5], swine infectious pathogens have greatly caught the attention of researchers from early 1990s when a lot of pig-producing countries experienced very huge economic losses as a result of emerging viral disease-causing agents such as* Porcine circovirus* type 2 (PCV2).

PCV2 has been known as a u niversal viral pathogen because of its presence in most, if not all the swine herds [6]. Its global prevalence and status have brought about its seropositivity rate of 20–80% in pigs coupled with very high incidence rate of 60% that is accompanied by general mortality rate of 3–10% and culling rate of 40% in seriously affected pig farms [7]. PCV2 is the main etiologic entity implicated in postweaning multisystemic wasting syndrome (PMWS) with other remaining* Porcine circovirus* associated diseases (PCVADs) [8]. It has also been established to be a necessary agent in the pathogenesis of PCVADs, but not a sufficient cause of the diseases that are known to be of great economic importance in pigs production worldwide [9, 10].

## 2. Historical Background, Classification, and Genomic Organization of* Porcine circovirus*

### 2.1. Overview of Initial Discovery and Subsequent Retrospective Studies of* Porcine circovirus* in Global Swine Herds

The initial discovery of* Porcine circovirus* (PCV) in Germany occurred in a continuous pig kidney cell line (PK-15 ATCC-CCL31) as a picornavirus-like contaminant and without any cytopathic effect, with initial assumption of having an RNA genome [11]. However, subsequent observations led to its description as a minute, nonenveloped, and icosahedral shaped virus possessing a genome with circular single-stranded DNA [12]. Experimentally, the PCV obtained from the PK-15 cell line did not produce any ailment in pigs [13, 14]. Subsequently in 1991 at Saskatchewan, Canada, another PCV emerged in a sporadic disease called PMWS which was characterized by weight loss, breathing discomfort, jaundice, and peculiar microscopic lesions in lymphoid tissues of infected pigs [15, 16].

Ellis [17] expressed the initial bias view of some investigators with “hope” of discovering a novel porcine lentivirus from the diseased pigs since they presented an AIDS-like syndrome. They hypothesized that a lentivirus caused the immunosuppression and allowed an observable, disease-causing proliferation of an endemically infectious agent proposed to be “new” circovirus [18]. However, the disease condition was further investigated by its first discoverers: Dr. John Harding, a swine consultant veterinarian in a private practice, and Dr. Edward Clark, a veterinary pathologist at University of Saskatchewan, Canada. Subsequently, a new DNA-virus with similar morphology to the PK-15 originated PCV was discovered from PMWS-affected pigs not only in North America but also in European countries [19–21] and has virtually been described in all continents of the world. Further examination of PMWS-associated PCV showed notable variations when compared with initially defined PCV [22, 23], and in a bid to differentiate the nonpathogenic PCV from pathogenic PMWS-associated PCV, the nonpathogenic strain was called* Porcine circovirus* type 1 (PCV1), while the latter was named* Porcine circovirus* type 2 (PCV2) [24].

Through PCV2 serological studies, it was observed that PCV2 infection is present globally, whereas the prevalence of its associated diseases is much lower; hence, the commonest form of PCV2 manifestation is the subclinical infection [25, 26]. However through various retrospective studies, the pathogenic PCV2 was observed to be in existence in swine herds from different regions of the world earlier before the PMWS outbreak in 1998 [27]. Jacobsen et al. [28] attempted to unravel the origin, spread, and pathogenesis of PCV2 and its diseases in northern Germany confirmed that the existence of PCV2 in pigs dated back to 1962 based on archived samples taken for necropsy within the period of 1961 and 1998 with the use of in situ hybridization techniques and polymerase chain reaction (PCR). Furthermore, it was discovered that the relative incidence of detectable viral nucleic acid and existence of PCV2-related lesions was significantly different by the subsequent years.

Total incidence of PCV2 infection was actually minimal within the period of 1961 to 1984, ranging from 0 to 11.7% but increased between 1985 and 1998 within the range of 14.3–53.3%. Also, PCV2-associated diseases were first seen in 1985 archived samples, while sequence analyses of some selected PCV2 DNA segments also showed high homology with currently existing PCV2 strains [28]. This retrospective study by Jacobsen et al. [28] is the foremost report on the detection of PCV2 in pigs worldwide. Many other retrospective studies had also confirmed the existence of the virus in swine from different countries prior to its official detection in 1998 (Table 1). However, associated diseases like PMWS and porcine dermatitis and nephritis syndrome (PDNS) were not diagnosed in the archived samples prior to 1985, implying that PCV2 infection solely is insufficient to bring about the onset of PCV2-associated diseases.

The overall epidemiological data so far thus shows PCV2 to have probably been in existence in the swine population across the universe for more than five decades [27–29].

### 2.2. Classification of Circoviruses

Historically, family Circoviridae comprises two genera, namely,* Gyrovirus *and* Circovirus* based on their morphology and genomic organization [30, 31], as established in the last published (ninth) report of International Committee on Taxonomy of Viruses (ICTV) of the year 2009 [32] with 11 species in the genus* Circovirus* which include* Canary circovirus, Pigeon circovirus*,* Duck circovirus, Finch circovirus, Goose circovirus, Beak and feather disease virus, Gull circovirus, Starling circovirus, Swan circovirus, Porcine circovirus type 1,* and* Porcine circovirus* type 2. Genus* Gyrovirus* consists of only* Chicken anaemia virus*, with different genome organizations compared to that of* Circovirus*. Due to the genomic organization and replication strategy of viruses listed in the genus* Circovirus, *they are known to have close relationship to the plant viruses called nanoviruses and geminiviruses, with characteristic stem loop structure situated at their origin of replication and the similarity of their replication proteins [33]. It had been asserted that circoviruses are the possible genetic intermediates between nanoviruses and geminiviruses [34].

However in a latest development, the taxonomy of Circoviridae has been revisited due to the discovery of new viruses and reevaluation of genomic features that characterize members of the family. In a current ratification by ICTV (2016), genus* Gyrovirus* has been reassigned to the family Anelloviridae while genus* Circovirus *and a new genus,* Cyclovirus,* were grouped together in family Circoviridae, consisting of twenty-seven (27) and forty-three (43) species, respectively [41]. Cycloviruses were discovered in 2010 as a group of viruses with very high relatedness to circoviruses, having genomic features that are closely related to them, and were tentatively named cycloviruses to differentiate them from the circoviruses [42, 43]. The establishment of the two groups in family Circoviridae becomes justifiable due to phylogenetic and genomic differences that exist between them vis-à-vis the host range differences [41].

### 2.3. Genomic Organization of* Porcine circovirus*

**Figure 1:**
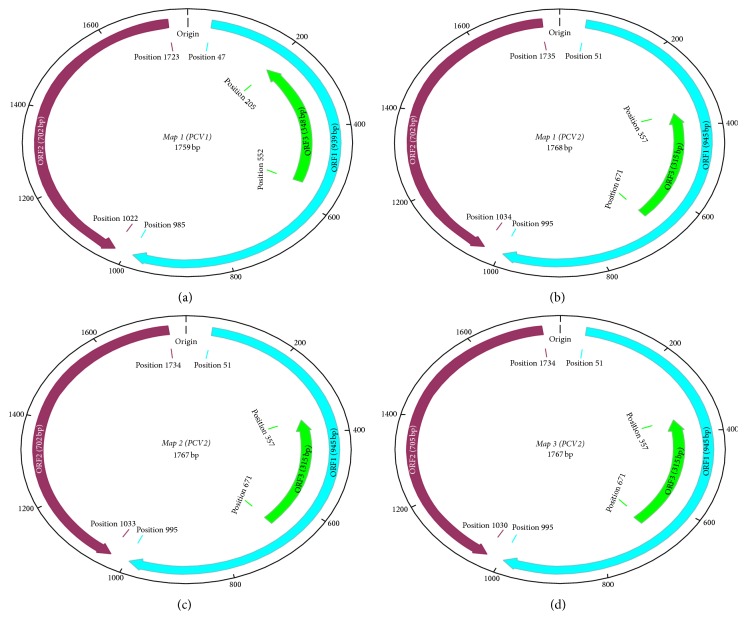
Genome organization of* Porcine circovirus* (NB: map (a) is for PCV1, while maps (b), (c), and (d) are for PCV2a and 2e; PCV2b and PCV2c and 2d, resp.), adapted from Zhai et al. [46].

Presently, PCVs are the smallest viruses found in animals. The diameter of their virions ranges from 17 to 21 nanometres in size [12]. Their genomes consist of single-stranded DNA that is circular in nature with about 1759 (PCV1) and 1767-1768 (PCV2) nucleotides sequence [12, 44]. PCV1 has only one genome map (Figure 1), which consists of two main open reading frames (ORFs) coding for replication initiating proteins and the structural capsid protein. The locations of the promoters of the genes (*Prep *and* Pcap)* have been determined. The capsid gene promoter is situated within the* rep *open reading frame (ORF1) precisely nucleotide position 1328 to 1252, while the replicase gene promoter is located at the intergenic area towards upstream of the replicase gene at nucleotide position 640 to 796 and forms a kind of overlapping at the origin of replication of PCV1 [45].

PCV2 genome organization is of three types: Maps 1 to 3 (Figure 1), corresponding to PCV2a and PCV2e; PCV2b; and PCV2c and PCV2d genotypes, respectively [46]. It also possesses three main ORFs: ORF1 (945 nucleotides at positions 51 to 995) which codes for replication proteins that controls replication process of the virus [47]; ORF-2 (702 or 705 nucleotides at positions 1734/1735 to 1030/1033/1034) which codes for the immunogenic structural protein (Cap) that determines the virus antigenicity [48]; and ORF3 (315 nucleotides at positions 671 to 357) which was reported to encode PCV2 protein that causes apoptosis in vitro and also involved in the pathogenicity of the virus in mice during an in vivo study [49, 50]. Furthermore, through a latest study by [51], a novel insight that gives deeper understanding of the biological function of PCV2 ORF3 was obtained when a nuclear export sequence (NES) was localized at the N-terminus of ORF3 that codes for protein which plays critical role in nuclear export activity. In another relatively recent study, a newly discovered viral protein from ORF4 (with 180 nucleotides on positions 386 to 565) was reported. The open reading frame is not really essential for PCV2 replication; however, according to He et al. [52], it has a function of bringing down caspase activity and also helps in CD4 (+) and CD8 (+) T-lymphocytes' regulation at the time of PCV2 infection.

## 3. *Porcine circovirus* Type 2 (PCV2)

### 3.1. Genotypic Classification of* Porcine circovirus* Type 2

**Figure 2:**
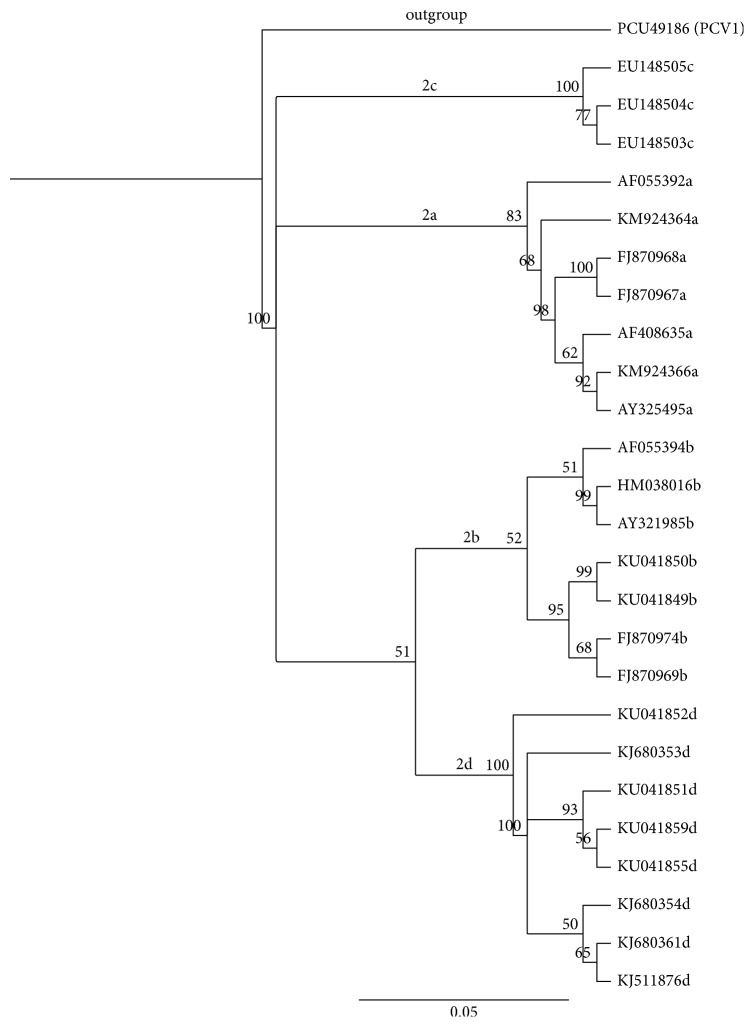
Four main genotypes of PCV2 as shown by phylogenetic tree that was constructed based on neighbor-joining method for complete genomes of some PCV2 sequences obtained from NCBI Genbank; genotypes of the virus were written on the main branches of the tree and PCV1 was used as outgroup (NB: the tree was constructed using Geneious 9.1.5, Biomatters Ltd. Boostrap values obtained from 1000 replicates are shown at the major nodes).

According to Ojok et al. [53], no virulence specific DNA polymorphism has been recognized in PCV2; however, characterization of the virus is important for epidemiological purposes. Based on phylogenetic studies and polymerase chain reaction-restriction fragment length polymorphism (PCR-RFLP), a prototype for classifying PCV2 was given that earlier divided the strains of the virus into five genogroups, namely, PCV2a to 2e [54]. However, through phylogenetic analysis that was based on cap gene and complete genome of PCV2 (Figure 2), the virus has been classified into four different genotypes which are PCV2a with five clusters, PCV2b with three clusters, PCV2c, and PCV2d [55–57]. PCV2a and 2b are known to have worldwide distribution, with PCV2b being the predominant genotype detected since 2003. The third type, PCV2c, was first reported in Denmark from an archived material [58] and, recently, it was discovered from live feral pigs in Brazil [59] and also from various field samples obtained from sick pigs in China [60]. The fourth genotype, PCV2d, was reported from China [56]; more recently it has been found dominating in most cases of PCV2 infections in the United State [61], South Korean pig population [62], and globally [63].

Based on latest argument of Chae [64], the classification of PCV2d is not in agreement with the standardized nomenclature rules for new PCV2 genogroups as stipulated by the European Union consortium on PCV-AD [57]. Classification of novel PCV2 genotypes is expected to fulfill two main requirements which are (a) having cut-off value on pairwise sequence comparisons (PASC) analysis of ≥0.0351 and (b)* p*-distance cut-off value of ≥0.035 [57, 65]. The* p*-distance that was obtained between PCV2b and PCV2d was 0.057 while the PASC value was just 0.020; hence, PCV2d according to Chae [64] does not meet up with the criteria by which it should be classified as a different genotype. As a result of this, it was proposed that PCV2d should be renamed as mutant* Porcine circovirus* type 2b (mPCV2b) on the account of the naming system laid down by the European Union consortium which serves to prevent any possible scientific confusion as regarding the PCV2 genotype names [64]. However, based on the* p*-distance value of 0.055 ± 0.008 between PCV2d and PCV2b that is greater than the earlier stated PCV2 genotype definition cut-off of 0.035, Xiao et al. [63] in a similar investigation, supported classification of PCV2d as an independent genotype without putting PASC cut-off value into consideration.

### 3.2. PCV2 Genotypes and Their Pathogenicity

It has been confirmed that both PCV2a and PCV2b are causing swine diseases with different levels of severity [66, 67], whereas PCV2c was discovered in Denmark from healthy pigs [58]. Earlier on before 2003, both PCV2a and 2b were usually prevalent in Europe and China, while in Canada and the United States, only PCV2a was common [68]. However, an observable spontaneous shift in the prevalence of PCV2a compared to PCV2b in commercial swine populations has occurred globally since 2003 with simultaneous increment in the severity of clinical PCVAD according to many available research findings [69–72]. Irrespective of the notable shift, however, there has not been any remarkable difference in pathogenicity observed between PCV2a and 2b experimentally [73] as their study using conventional specific-pathogen-free (SPF) pig model observed no appreciable difference in the virulence of the two viruses in pigs that were experimentally infected with them.

Also in a similar study conducted by Lager et al. [74] using germ-free experimental pigs that were infected with PCV2a or PCV2b infectious clone, it was reported that the pathological manifestations and viral antigen load observed in the two treatments were not really different, even though the pigs that were infected with PCV2b had quicker onset of diseases and higher overall morbidity/mortality of 100% than those infected with PCV2a which was just 25%. Similarly, in a meta-analysis experiment which focused on determining the contributory factors that culminate in development of PMWS experimentally, it was observed that inoculating the pigs with PCV2b among four other factors favoured more successful reproduction of PMWS in the pigs [75]. However, the fact remains that the disease had been successfully induced in healthy pigs using either PCV2a or 2b, though, there is possibility for the observable difference in virulence between the two main genogroups, to be a function of one among several other potential factors that influence the development of the PCVADs [70].

Notwithstanding several arguments and counterarguments on virulence potentials between the two major genotypes, series of findings have shown that there is no similarity in the antigenic composition of the two genotypes [76, 77]. Sequences disparities that exist between them are usually seen at the viral structural capsid gene as well as a specific signature sequence motif that differentiates the two were observed in the genomes of the two major genotypes of the virus [78, 79]. Furthermore, reported isolation of more virulent recombinant PCV2b strains (mPCV2b) from cases of vaccine failure from countries like China [56, 80] and United States of America [81, 82] and recently from Republic of South Korea [83], Brazil [84], Uruguay [85], and Germany [86] has become a serious cause for concern due to the corresponding increase in virulence and very fast spread. Series of latest findings on the escalation of genetic differences in PCV2 according to Constans et al. [69] and subsequently confirmed by Reiner et al. [87] suggests that current vaccines that are based on PCV2a may be the driving force behind the viral selection and evolution with resultant emergence of more virulent PCV2b strains as reported in cases from fields.

### 3.3. Pathology, Concurrent Infections with Other Pathogens, and Transmission Modes of PCV2 and Its Associated Diseases

#### 3.3.1. Clinical Pathology of PCV2 and Its Associated Diseases

PCV2 and its associated diseases primarily affect pigs in their late nursery stage and growers, ranging between 7 and 16 weeks old due to presumed protective influence of maternal antibody in younger piglets [88]. Clinicopathological manifestations of PCV2 infection are wasting (i.e., loss of weight), unthriftiness, skin paleness, jaundice, enlarged lymph nodes, and diarrhoea [25, 89, 90]. PCV2 majorly has predilection for the immune system of pigs by preferentially targeting the lymphoid tissues, leading to its depletion and histiocytic replacement in them which are the observable typical histological lesions [26, 91]. Pathological conditions could be exacerbated by immunostimulation or coinfections with other pathogens which occur as a result of immunosuppression and immunity reduction in the affected pigs [7, 90].

According to Segalés [26], the PCV2 infection's clinical scope and pathology have greatly increased over the years. Apart from the most popular and ravaging PMWS, other disease conditions and disorders which include reproductive failures which is usually characterized by abortions or stillbirths with foetuses having an observable necrotizing myocarditis have been observed in the field cases of PCV2 infection and have been reproduced experimentally [92, 93]. Moreover, a respiratory disease which is termed porcine respiratory disease complex (PRDC) which is known with respiratory distress has also been discovered in pigs infected with PCV2, with observable bronchiolitis with mononuclear infiltration of the lungs and interstitial pneumonia [94, 95]. The pathogen has equally been observed to be in connection with PDNS which is a disease that causes formation of skin lesions of red to purple colour in affected pigs. It is also characterized by glomerular and interstitial nephritis and vasculitis [96, 97], although it has not been experimentally reproduced in pigs infected by PCV2 [90]. The list also includes enteritis and proliferative and necrotizing pneumonia (PNP) [25, 98]. Another disease condition in which PCV2 was initially implicated is congenital tremor type A2 [99], which some subsequent research findings later exonerated from being the culprit etiologic agent [100, 101]. Nevertheless, in a latest research work, PCV2 antigen was again found in the brain tissue of newly born piglets having congenital tremor [102].

#### 3.3.2. Multifactorial Status of PCVADs

Though PCV2 had been confirmed to be the primary pathogen implicated in PCVAD, under experimental conditions, it has been established that infection with PCV2 solely in most cases does not cause overt clinical disease. Based on available information, it has been shown that several infectious cofactors and noninfectious conditions such as concurrent infection with other pathogens [103, 104], host genetic make-up [105], and management practices [106] are crucial for disease progression to PCVAD. Consequently, infections with PCV2 have been regarded as multifactorial at the instances of other underlying cofactors that exacerbate infection with the viral pathogens resulting in the clinical disease manifestations. Infectious cofactors that have been extensively studied till date consist of* Porcine parvovirus*, PPV [107–109],* Porcine reproductive and respiratory syndrome virus*, PRRS [110],* Swine influenza virus* SIV [111],* Torque teno virus* [71],* Swine hepatitis E virus*, HEV [112],* M. hyopneumoniae *[111, 113]*, Salmonella *spp. [114],* E. coli* [115], and* Metastrongylus elongatus* [116]. The noninfectious cofactors include the genetic background of the pig, high stocking density, and prevailing environmental conditions such as temperature fluctuation within the pen [106].

#### 3.3.3. Transmission Modes of PCV2 Infection

PCV2 is a pathogen with multidimensional spreading potentials. The rate at which the viral pathogen spreads brought about the analogy: “spreading like a wildfire” according to Meng [117] in a Guest Editorial Review. The spread of PCV2 can be viewed within the scope of international transmission through trading of pigs and pig products and also transmission among local herds. PCV2 has been confirmed to be transmitted through trading of live pigs with subclinical infection due to the subtle nature of the disease, which invariably calls for a more critical diagnostic check on imported animals or their products [5, 118]. There have been reported cases of transmission through selection of boars for breeding purposes and the importation of semen for the purpose of artificial insemination [119–121].

Spreading of PCV2 among local herds has been reported in various dimensions ranging from pig to pig [122, 123], man to pig [124], rodents to pig [125], insects to pig [126, 127], and pen environment to pig [128]. This becomes possible due to numerous routes of shedding the virus in cases of systemic infections, as observed in both natural and experimental conditions including oronasal secretions, faeces, urine, colostrum, milk, and semen from infected boar as earlier mentioned [129–132].

### 3.4. Diagnosis, Prevention, Control, and Treatment of PCV2 and Its Associated Diseases

#### 3.4.1. General Diagnosis of PCV2

As a complex disease of diverse clinical signs, accurate diagnosis of PCV2 infections is highly important in order to implement appropriate intervention strategy on affected herds [66]. Diagnosis of PCV2 infections could be done basically in two major ways. Firstly, on the basis of clinical disease manifestations, which could be regarded as tentative, and, secondly, based on the confirmatory detection of PCV2 antigen in lymphoid tissues and organs such as the liver, lungs, kidney, or intestine of an infected animal [89]. According to Gillespie et al. [89], for a farm to be tagged as experiencing PCVAD disease, percentages of clinical signs could be seen as follows: loss of weight (98.1%), diarrhea (77.2%), lymphadenopathy (44.8%), dyspnea (75.1%), central neurologic signs (39.6%), inappetence (90.4%), jaundice (37.1%), and death (96.8%). However, in a situation whereby a farm is experiencing subclinical infection, true status of such herd as regarded to PCV2 infection could be mistaken if based on clinical signs manifestation. This is because majority of clinically healthy pigs could be seropositive meaning subclinical infections [89].

PCV2 antigen or nucleic acid detection in samples from a swine herd is known as the golden standard suitable for the confirmatory diagnosis of PCVAD. This has been effectively achieved through PCR, immunohistochemistry (IHC), and in situ hybridization (ISH) [66, 133]. Other diagnostic tests that have been developed and used in PCV2 detection in infected pigs include enzyme linked immunosorbent assay, immunofluorescence assay, IgM immune-peroxidase monolayer assay, serum virus neutralization assays, virus isolation, and electron microscopy [66, 134, 135].

#### 3.4.2. Prevention, Control, and Treatment of PCV2 till Date

As a multifactorial disease which has been linked to both infectious and noninfectious factors, effective control of PCVAD can be solely achieved not only by vaccine applications but also by preventing triggering factors through improved swine management, control of coinfection, and change of genetic background of pigs through careful selection of boar for breeding. Though vaccination is traditionally considered as the most effective method for preventing viral diseases, it had been established that the protection period of vaccine against the disease is limited and that complete eradication of the virus has not be achieved through vaccination [136].

However, the currently available commercial PCV2 vaccines (Table 2), all of which are either an inactivated whole virus vaccine or subunit vaccine designed base on the immunogenic Cap protein of PCV2a, have shown effectiveness against clinical disease expression and enhanced major production parameters (mortality and average daily gain) in swine herds with PCV2 occurrence. While vaccine applications did not outrightly prevent infection with PCV2 nor restrict its spread, a reasonable reduction in the systemic viral loads and shedding had been observed, which invariably help in decreasing the load of the virus in the environment [137–139]. Although, animals under vaccination could still be infected with the virus, they generally do have lower viral loads compared to those not vaccinated. Thus, currently available vaccines applied most especially as a single-dose protocol do not give sterilizing immunity in swine herds [140].

Moreover, the introduction of the PCV2a vaccines has been observed to cause a corresponding world-wide shift in the prevalence of genotypes from PCV2a to 2b with attendant severe clinical manifestations in vaccinated herds [141]. Also, some reports on PCVAD cases have shown that a new variant named mutant PCV2b (mPCV2b) was found in diseased pigs despite their prior vaccinations [142]. This has brought about serious concerns on possible emergence of PCV2 vaccine escape strains; however, it was observed that one of the current vaccines was effective against mPCV2b infection under experimental setup [143]. Nevertheless, it has been asserted that series of recent research findings concerning the increased PCV2 genetic diversity connotes that the available vaccines which are based on PCV2a may be inducing the observable selection pressure and possibly be the driving force behind the viral evolution [69], being pivoted on observable high nucleotide substitution rate earlier suggested for the continuous evolution of PCV2 and the emergence of novel PCV2 strains [144].

According to Constans et al. [69], some immune cells epitopes that were known to elicit immune response in the vaccine strain were not seen in the field strains, showing that there is a silent change in the antigenic profile of the strains as many nonconserved epitopes have been predicted to have immune cells functions. Furthermore, the substitutions in the epitopes have been ascertained to affect the immune response greatly, thereby causing immune escape [69]. Hence, many calls and recent research findings have raised support for a rational development of PCV2 vaccines to target evolving genotypes in order to increase the current threshold of protection against PCV2 and its associated diseases [69, 142, 145].

Based on recent laboratory findings of Peng et al. [146] there is a future possibility of producing effective commercial PCV2 antibodies and vaccines that could be based on nonvariable* rep* protein compared to the presently available ones that are based on variable* cap* protein of the virus. From the study, it was observed that the recombinant plasmids of* rep* gene that was constructed shows an efficient expression in the prokaryotic system and also the engineered proteins were immunogenic. Furthermore, the characterized polyclonal antiserum made with* rep* protein shown good reactivity and displayed considerable specificity against PCV2 in PK-15 cell line. Hence, the Rep protein seems to be having future potentials in PCV2 antibody and vaccine development [146].

The utility of commercially available PCV2 vaccines (both inactivated and subunit vaccines based on PCV2a genotype) has been proven over the years as they have shown effectiveness in decreasing mortality and increasing growth parameters in commercial swine herds [67]. There are unquestionable evidences from accumulated field data that confirms the efficacy of the commercial vaccines when production parameters such as average daily weight gain and economic gains in vaccinated pigs were compared with the unvaccinated pigs. In fact, cross-protection against other coinfecting agents is an additional advantage in the use of PCV2 vaccines [67, 147]. The good news is that, in countries where vaccination has been grossly employed, there has been an appreciable declination in the prevalence of PCV2 with good vaccination practices.

However, despite the huge successes recorded so far on PCV2 vaccines, it has been asserted that the protection period of a PCV2 vaccine against the disease is limited and that the virus could not be eradicated by a mass vaccination procedure when applied for a period of one year. This was because, after some months of stopping the intensive vaccination programme, there was a reemergence of PCV2 infection [136]. Although vaccination is recommended for healthy pigs, it is ineffective for pigs that are already infected with PCV2 [148]. In addition, the inherent potentials of vaccines to cause viral evolution [149], coupled with ineffectiveness of vaccines to prevent the multifactorial disease such as PCVAD, have necessitated urgent discovery and development of safe drugs as alternatives in a bid to eradicate or control PCV2 [150].

#### 3.4.3. Promising Efforts on Ethnobotanicals

Recently, research efforts have been focused on developing antiviral drugs from natural compounds with promising outcomes. In a recent study in which twenty natural compounds isolated from traditional Chinese plants were evaluated for their antiviral activities against PCV2 in vitro, it was observed that Matrine (an alkaloid compound purified from the dried roots of* Sophora flavescens* Ait) and Scutellarin (a flavonoid compound from* Scutellaria barbata* D. Don) showed appreciable inhibition rates of 57 and 72.69%, respectively, against the virus out of all the tested compounds [148]. Furthermore, in a more recent study aimed at exploring the antiviral potential of Matrine against PRRSV and PCV2 concurrent infection in a porcine alveolar microphages (PAM) cell model, it was observed that the use of Matrine abated the proliferation of PRRSV and PCV2 effectively at twelve-hour postinfection period. This finding further necessitates the need for immediate exploration of natural products such as Matrine as antiviral agent against PCV2 in clinical settings [150].

In another related study which is aimed at investigating the antiviral activity of a phenylpropanoid dibenzylbutyrolactone lignan called Arctigenin (ACT), extracted from another Chinese traditional herb named* Arctium lappa *L. against PCV2 in vitro and in vivo, another promising result was obtained. It was observed that dosage of 15.6–62.5 *μ*g/mL of ACT efficiently inhibit the thriving of PCV2 in PK-15 cells, while the intraperitoneal injection of 200 *μ*g/kg of ACT into PCV2-challenged mice significantly inhibited PCV2 proliferation in the lungs, spleens, and inguinal lymph nodes of the mice, showing similar effect to ribavirin, an antiviral drug that was used as positive control, thereby demonstrating the effectiveness of ACT as an antiviral agent against PCV2 both in vitro and in vivo [151].

## 4. Global Status of PCV2 and Its Associated Diseases: Sub-Saharan Africa Scenario

### 4.1. PCV2 and Its PMWS: Is the Global Disease a Global Concern?

Sequel to the first known outbreak and description of porcine multisystemic wasting syndrome, an important PCVAD in a very healthy, farrow-to-finish swine farm situated in Northeastern Saskatchewan, Western Canada, North America, in early 1990s [15, 152, 153], PMWS and other PCVADs have been subsequently seen in all regions of the world including many European countries such as Spain [21], France [154], Hungary [155], and United Kingdom [19, 36]; South America countries such as Brazil [156] and Uruguay [157]; Asian countries such as Korea [158] and China [159]; Oceania, Australia [160]; Caribbean, Cuba [161]; Middle East, Israel [162]; and African countries such as South Africa [119] and, recently, Uganda [53]. The ubiquitous status of* Porcine circovirus* and its numerous associated diseases has been said to be linked to the marketing of subclinically infected pigs vis-á-vis the choosing of such pigs for breeding programmes as the virus is known to be spread through semen from boars [5, 163].

Vidigal et al. [5] reported significant link between the routes of dispersal of PCV2 and international marketing of live pigs from obtained data, thereby showing how important the movements of livestock around the globe could be in the emergence and spread of new pathogens. Therefore, increase in the global trade of livestock and their products and increase in global livestock production as a result of the use of intensive animal rearing system have arguably contribute to the spread of infectious pathogens globally [5, 149, 164]. In addition to those mentioned facts and the multiple transmission routes earlier described for PCV2 coupled with the long-lasting viral life, the significance of world-wide proliferation of PCV2 in influencing the PMWS epidemics is indicated [27, 144, 165].

### 4.2. Incidence of* Porcine circovirus* Type 2 in Swine-Producing Countries of Sub-Saharan Africa

#### 4.2.1. First Emergence of PCV2 in South African Swine Herds

Drew et al. [119] reported what seemed to be the first cases of PCV2 associated diseases in the region, which occurred in Gauteng Province, South Africa. The cases occurred in June 2001 on a large, well-managed breeding unit which serves as supplier of breeding stock to three member farms, separated by distances 100 and 500 km apart. The initial clinical manifestation observed in the pigs was PDNS which affects young pigs of two to three months old. In no distant time, the expression of clinical signs associated with PMWS became more evident, to the extent that by November 2001, the morbidity due to PMWS had increased to about 30 to 40% with mortality remaining below 10% [119].

Moreover, clinical manifestations of PMWS were also seen in approximately the same percentage of pigs of the same ages bred on one of the member farms, specifically, the one situated about 100 km away from the main farm after the introduction of pigs from the main farm, on which the disease had first been noticed. However, tissues were collected from just two affected animals of about 12 to 15 weeks of age from each of the two premises and were submitted for histological, immunohistological, and molecular analysis. Tissues sections from the spleen, liver, brain, and heart of the four pigs did not show significant histopathology. It was observed that the lesions indicative of PMWS were concentrated in the precapsular, bronchial, and gastrohepatic lymph nodes. Also in many subcapsular areas in the lymph nodes, there were widespread areas of diffuse lymphocyte depletion with presence of multinucleate giant cells as evidence occasionally [119].

Furthermore, the molecular analysis of a 501-nucleotide fragments of the viral genome amplified from tissues samples of the reported cases revealed that they were identical to a PCV2 isolate from Iowa, USA, with GenBank Ascension number* AF264039* [166], having just only two nucleotide substitutions from the USA isolate. The sequence obtained from a lymph node of a piglet from the main farm yielded the complete PCV2 genome (1768 base pairs) and was designated SAI, this is the only isolate deposited so far in the GenBank with ascension number* AY325495* from South Africa and virtually in the entire sub-Saharan Africa. In their conclusion, Drew et al. [119] recommended for further molecular epidemiological studies to investigate PCV2 at other sites in South Africa. However till this time, little or no work has been done to that effect in South African pig population.

**Figure 3:**
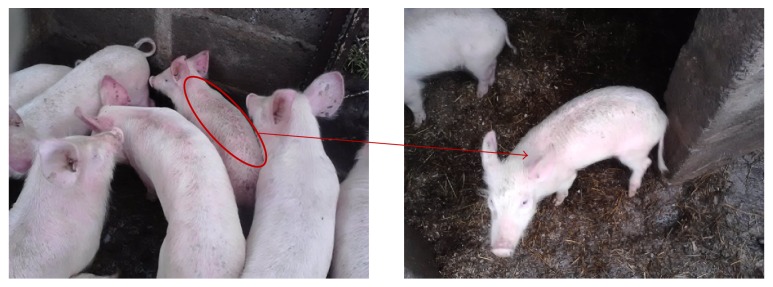
Wasting appearance of a pig with a notable respiratory distress within a herd of the same age at a farm in Lukhanji Local Municipality, Chris-Hani District, Eastern Cape, South Africa (Picture taken in April, 2016 during a sampling exercise).

This assertion is further confirmed by more recent research work of Mokoele et al. [167] in which it was categorically stated that “though PCV2 may be an economically important disease in South Africa, to date, no specific surveillance has been conducted to validate the current status because the disease is thought to be ubiquitous in most countries.” However, seeming negligence to know the health status of swine herds of the country as regarding the ubiquitous viral pathogen could pose a serious problem for the industry in a nearest future due to a recent observation during a field trip which was part of our ongoing research work that is focused on determining the occurrence of some RNA and DNA viruses in swine herds of Eastern Cape Province South Africa; pigs with typical clinical manifestations of PMWS were seen (Figure 3), showing likelihood of existence of the viral pathogen in pig herds of the country.

In another study by An et al. [168], nucleotide sequences of 197 PCV2 strains submitted to GenBank nucleotide database at the National Center for Biotechnology Information (NCBI) from all over the world and 36 PCV2 strains obtained from PMWS and PDNS cases in Korean pigs over an 8-year period were used in grouping PCV2 into two groups (1 and 2) by phylogenetic tree analysis and multiple alignments of nucleotide sequences. In their study, it was observed that three countries, namely, South Africa, United Kingdom, and Thailand, were having just one PCV2 complete genome sequence each on GenBank as far back as then, indicating dearth of information on PCV2 from the three countries. They recommended further studies on PCV2 from the countries so that accurate documentation of PCV2 strains circulating in the three countries could be done. However till date, nothing has been done in South Africa as regarding the clarion call.

#### 4.2.2. PCV2 in Ugandan Pigs

Recently, Ojok et al. [53] also reported on the molecular detection and characterization of PCV2 from pigs in Uganda, however with limitation of using a relatively small sample size (*n* = 35) like that of Drew et al. [119]. Only three cases of PCV2 were found in their study and they also recommended that further studies be conducted so as to fully understand the true prevalence of the virus in swine population of Uganda as well as their genetic diversity. The three PCV2 sequences in their study were observed to cluster with PCV2b genotype which was originally referred to as the European cluster or PCV2 group 1 in contrast to the South African strain which clustered with the PCV2a previously referred to as the North American strains or PCV2 group 2.

In a similar unpublished study conducted by Jonsson [169] aimed at investigating the disease transmission patterns in the livestock-wildlife interface in Uganda, being part of the Emerging Infectious Diseases (EID) surveillance programme conducted to study the prevalence of PCV2 in domestic pigs in Uganda, ninety-one domestic pigs around Murchison Falls national park were sampled and analysed. The sampled domestic pigs were all negative for PCV2a, while for PCV2b which is known to be genogroup mostly associated with PMWS, a point prevalence of 77% was reported. This is in support of the findings of Ojok et al. [53] that reported the presence of PCV2b genotype in a separate study on Ugandan pigs. The point prevalence of 77% for PCV2b in the study cannot be generalised to all of Ugandan pigs; this is because the sample selection was too small to arrive at such conclusion. As a result of this, more extensive studies were recommended by Jonsson [169], so as to obtain more accurate data on the prevalence of the PCV2b in Ugandan pigs.

#### 4.2.3. PCV2 till Date in Cameroonian Pigs

The study of Ndze et al. [170] is another recent effort aimed at describing the occurrence and genetic diversity of selected DNA viruses belonging to different families, namely, Adenoviridae, Circoviridae, Anelloviridae, and Parvoviridae in Cameroonian pigs. However, only viruses belonging to the family Parvoviridae were detected, most especially those within the bocaviruses. In their remarks, they attributed their failure to detect other groups of viruses including* Circovirus *which is known to be ubiquitous to several possible factors including but not limited to short sampling period and low study sample number.

## 5. Conclusion

So far, the status of porcine circovirus type 2 is practically unknown in pigs of many swine-producing countries in sub-Saharan Africa. Moreover, countries where some research works have been done till date are having insufficient data that could enhance the detailed characterization of the viral genogroup that may likely be in circulation in swine herds of many countries within the region. There is, therefore, an urgent need for large scale molecular epidemiological studies on the virus and its associated diseases in the region, including origins of observed genogroups. This will help in facilitating adequate preventive and control measures against the pathogen through the establishment of effective vaccination regime that could help in combating this globally important and emerging porcine viral pathogen with huge economic implications in the global pig industry.

## Figures and Tables

**Table 1:** Retrospective studies on earlier occurrence of PCV2 in pigs from some countries of the world.

Country	Sampling period	Reference
Northern Germany	1961–1998	[28]
Belgium	1969	[35]
UK	1970–1997	[36]
Northern Ireland	1970-1971	[37]
Northern Ireland	1973–1999	[38]
Canada	1985	[39]
Spain	1985–1997	[40]

**Table 2:** Available commercial PCV2 vaccines and some of their features.

Vaccine	Antigen	Recommended usage	Manufacturer	Adjuvant
Circovac®	Inactivated PCV2a virus (whole virus)	Breeding sows (two doses at 5 weeks of age and 2 weeks ante -partum)/piglets	Merial, Lyon, France	Mineral oil
FosteraTM PCV	Killed chimeric PCV1-2a virus	One dose for piglets of three weeks old or above	Pfizer	SL-CD aqueous
Ingelvac CircoFLEX®	Capsid protein of PCV2a (recombinant)	One dose for piglets of three weeks of age or older	Boehringer Ingelheim Vetmedica Inc. Missouri, U.S.A.	Carbomer
Circumvent® PCV	Capsid protein of PCV2a (recombinant)	Two doses at 3 and 6 weeks of age of piglets/growers respectively	Intervet/SP (Merck)	Microsol Diluvac Forte® (MDF)
Porcilis® PCV	Capsid protein of PCV2a (recombinant)	One dose for piglets of three weeks of age or older	Schering-Plough (Merck)	Mineral oil
